# A Comparative Clinical Study of the Effect of Denture Cleansing on the Surface Roughness and Hardness of Two Denture Base Materials

**DOI:** 10.3889/oamjms.2016.089

**Published:** 2016-08-22

**Authors:** Amani Ramadan Moussa, Wessam Mohamed Dehis, Asmaa Nabil Elboraey, Hisham Samir ElGabry

**Affiliations:** Prosthodontics Department, National Research Centre, Giza, Egypt

**Keywords:** Clinical study, complete denture, denture cleansing, denture base roughness, denture base hardness

## Abstract

**AIM:**

This study aimed to verify the influence of oral environment and denture cleansers on the surface roughness and hardness of two different denture base materials.

**METHODS:**

A total of sixteen identical removable disc specimens (RDS) were processed. Eight RDS were made from heat-cured acrylic resin (AR) and the other eight were fabricated from thermoplastic injection moulded resin (TR). Surface roughness and hardness of DRS were measured using ultrasonic profilometry and Universal testing machine respectively. Then the four RDS (two AR and two of TR) were fixed to each maxillary denture, after three months RDS were retrieved. Surface roughness and hardness of RDS have measured again.

**RESULTS:**

The surface roughness measurements revealed no significant difference (p >0.05) for both disc groups at baseline. However, both groups showed a significant increase in the surface roughness after three months with higher mean value for (TR) group. On the other hand, the (AR) group showed higher hardness mean value than (TR) group at baseline with no significant decrease in the hardness values (p >0.05) following three months follow-up period.

**CONCLUSIONS:**

Denture cleansers have an effect on the denture’s surface roughness and hardness concurrently with an oral condition which will consequently influence the complete dentures’ lifetime and patients’ satisfaction.

## Introduction

Polymethyl methacrylate (PMMA) resin has a long, clinically established history for being utilised as denture base material, owing to its excellent aesthetic, adequate physical properties, reasonable coast and easy processing technique [1-3]. However, dimensional inaccuracies, microbial adhesion, inadequate mechanical properties and allergic side effects are the greatest disadvantages that affect the clinical performance of PMMA prosthetics [4]. Continuous research focusing on PMMA properties improvement has led to the emergence of new processing techniques and alternative polymeric materials known as thermoplastic resins. These materials exhibit high creep and solvent resistance, excellent wear characteristics and high fatigue endurance. In addition, they have very little or almost no free monomer; therefore, they offer another option for allergic patients. Among thermoplastic resins is PMMA based resin which is used as denture base for both removable and complete dentures [5, 6].

Clinically, dentures are exposed to temperature variations during smoothening and polishing procedures at the time of construction. In the oral environment, dentures are also subjected to thermal alterations through food intake, besides the unavoidable biofilm development and bacterial colonisation on denture surfaces [7]. This colonisation is an important stage in the pathogenesis of denture stomatitis and other diseases not only for elderly and immune-compromised patients but also for healthy individuals [8].

While the surface roughness of the denture base is a contributing factor for bacterial colonisation, the adhesion of microorganisms to a surface is a prerequisite for its colonisation [9-11]. Furthermore, hardness is another property that influences the surface characteristics of denture base material as it facilitates the prosthesis finishing and maximises its resistance to abrasion and scratching during service and cleansing [12, 13]. Nevertheless, the maintenance of denture hygiene and effective microbial film removal represent an essential demand for denture wearers’ health. Currently, a number of mechanical and chemical denture cleansers are available. The mechanical method involves brushing with a dentifrice or neutral soap [14, 15]. While in the chemical method, dentures are immersed in products containing chemical agents as alkaline hypochlorite solution and alkaline peroxides (oxygenated cleansers). The latter is safe, easier and frequently utilised procedure, particularly in old aged patients. Beside their chemical efficiency against biofilm, they also eliminate stains mechanically by liberating oxygen [16, 17]. In literature, both the surface roughness and hardness have been widely studied in-vitro; however, no in-vivo reports are available about the effect of oral environment together with denture cleanser. Accordingly, it was interesting to verify the influence of proceeding factors on the surface roughness and hardness of two denture base materials in-vivo.

## Materials and Methods

### Construction of removal discs’ specimens (RDS)

**Figure 1:**
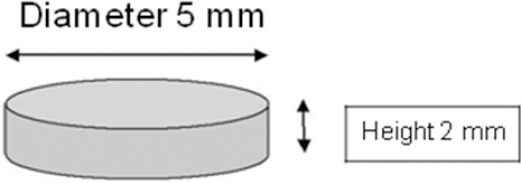
Diagrammatic representation for the circular disc

The two different denture base materials used in this study are listed in Table 1. A total of sixteen identical disc specimens (5 mm. in diameter and 2mm. in thickness) were processed (Fig. 1). Eight were made from heat cured acrylic resin (AR) and the other eight were fabricated from thermoplastic injection moulded resin (TR). All specimens were produced in moulds prepared by insertion of stainless steel rings into the metal dental flask filled with type III dental stone [18, 19]. After complete stone setting, each RDS denture base material was proportioned, mixed and processed according to each manufacturer’s instructions shown in Table 1. Then the flasks were allowed to bench cool and the specimens were removed.

**Table 1:** Denture base resins and their processing techniques

Denture resin	Processing type	Polymerization procedure	Powder/Liquid Ratio-
Acrostone	Heat activation (WHW plastic, England Packed by Anglo-Egyptian Lab)	Pack and press fast heat curing at boiling water 100 for 20 min	21/6 ml
Bre-Crystal (bredent- Germany)	Heat activation	Injection-molding 260°C for 26 min. Pressure: 5 bar	Single Component

For TR specimens, spruces were carefully removed with tungsten carbide burs (Bre-dent, GmbH & Co.KG Germany).

AR and TR disc specimens were finished and polished using medium and fine grit acrylic polishers

(Bre-dent, GmbH & Co.KG Germany). Finally, all RDS were cleaned and disinfected utilising denture cleansing tablets (Protefix, Queisser, Germany) and stored in distilled water to measure both surface roughness and hardness.

### Dentures construction and specimens’ fixation

Four edentulous male volunteers, aged 50-60 year, willing to have a new set of complete dentures, participated in the current clinical study. Patients were selected from National Research Center (NRC) dental clinic fulfilling the following criteria:

- Healthy firm mucoperiosteum without any signs of inflammation or flabby tissues.
- Patients were free from any systemic and neurological diseases that might affect their ability to co-operate, follow the recommendations and instructions of the clinician.
- Smokers were not included in the study.

Each participant signed a written informed consent before sharing in this study. The study protocol was approved by the ethics committee at NRC.

Complete Dentures were constructed and processed using conventional heat cured acrylic resin (Acrostone, WHW plastic, England Packed by Anglo Egyptian Lab) following the manufacturer’s instructions. The maxillary dentures were prepared to receive the four RDS specimens by creating two circular holes on either side of the midline at the denture’s flat palatal portion using tissue bunch with 6.1 mm diameter (Leader, Italy). Then, dentures were replaced with a replica of the master cast to facilitate fixation of the specimens, where two RDS of each denture base material were fixed on each side using self-cured acrylic resin (Acrostone, WHW plastic, England Packed by Anglo Egyptian Lab).

**Figure 2:**
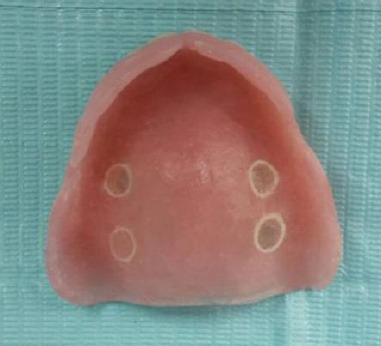
Maxillary complete denture with fixed RDS

A fine acrylic polisher (Bre-dent, GmbH & Co. KG ·Germany) was used to eliminate any irregularities or excess of self-cured resin (Fig. 2). Finally, the dentures were disinfected by means of cleansing tablets (Protefix, Queisser, Germany) and stored in room temperature tap water until delivery time.

Dentures were delivered to patients and they were instructed to maintain strict denture hygiene measures using cleansing tablets (Protefix, Queisser, Germany) once a day for 3 months.

### Retrieval of Removable Acrylic Resin Specimens

Patients were recalled after 3 months where the maxillary dentures were removed and gently cleansed with soft denture brush to remove any gross soft deposits. The dentures were disinfected with the same given denture-cleansing agent before retrieving of RDS. Then RDS were retrieved with tissue bunch 6.1 mm diameter (Leader, Italy). The holes in maxillary dentures were restored with heat-cured discs using self-cured acrylic resin. The retrieved disc specimens were disinfected once more and stored in tap water for one day before surface roughness and hardness were measured.

### Measurements of Surface Roughness and Hardness

#### Measuring Surface Roughness (μm)

Surface roughness in terms of roughness average (Ra) was estimated by the National Institute for the Standards-Egypt using ultra-sonic profilometer (form Talysurf i200, Taylor Hobson-AMETEK’s, USA). The first surface roughness readings were measured immediately after specimens’ preparation as a baseline record and the mean of three readings was enrolled. The final roughness measurements were done after 3 months of RDS retrieval.

#### Measuring Surface Hardness (kg/mm)

The hardness was measured using the Universal testing machine (Nexus 4503, INNOVATEST, Netherlands, Europe) in the National Research Centre, with Vickers diamond indentator. A 100 g load was applied for 10 seconds with 20 x magnification. Every specimen was subjected to three indentations (one on the centre, two on the border) and the average value was recorded for each RDS material. Similarly to roughness, the first hardness readings were achieved immediately after specimens’ preparation and the final hardness measurements were carried out after 3 months of RDS retrieval.

### Statistics

Data were analysed using IBM^®^ SPSS^®^ (SPSS Inc., IBM Corporation, NY, USA) Statistics Version 23 for Windows. Independent t-test was performed to compare the influence of denture cleansing tablets and oral environment on both the surface roughness and hardness of two different denture base materials utilising removable discs’ specimens (RDS) fixed to maxillary complete dentures. The significance level was set at *p* ≤ 0.05.

## Results

Table 2 and 3 represents the mean and standard deviation (SD) values for the two denture base materials RDS; heat cured acrylic resin (AR) and the thermoplastic resin (TR) prior to fixing it to dentures and 3 months following insertion and utilising using of cleansing tablets.

**Table 2:** Roughness measurement (*μ*m) for heat cured (AR) and thermoplastic resin (TR) DRS before and 3 months following the use denture cleansing tablets

		Denture Base				P value

		Heat Cure AR		Thermoplastic resin		
		Mean	SD	Mean	SD	
Roughness	Before	0.20	0.08	0.26	0.08	0.06 NS
	After	0.35	0.11	0.37	0.11	0.723 NS
p-value		0.001*		0.023*		

Note: Means with the same letter within each row are not significantly different at p ≥ 0.05.

* = Significant, NS= Non-significant.

**Table 3:** Measurement of surface micro hardness (Kg/mm^2*)* for heat cured (AR) and thermoplastic resin (TR) before and 3 months following the use denture cleansing tablets

		Denture Base				p-value
		Heat Cure AR		Thermoplastic PMMA		
		Mean	SD	Mean	SD	
Micro hardness	Before	18.46	2.08	13.65	2.01	0.006*
	After	14.90	4.20	11.81	.53	0.192 NS
p-value		0.151 NS		0.123 NS		

Means with the same letter within each row are not significantly different at p≥0.05.

* = Significant, NS= Non-significant.

Despite the lower mean roughness value of heat cured resin compared to thermoplastic resin (0.20 *μ*m & 0.35 *μ*m respectively) as shown in Table 2; the surface roughness measurements revealed no significant difference (*p* > 0.05) both before and after fixation of RDS and the use of cleansing tablets for both RDS materials. Moreover, each RDS material showed a significant increase (*p* < 0.05) in the surface roughness after three months (Table 2) with higher mean value for TR than AR. (0.37 *μ*m & 0.35 *μ*m respectively).

Comparison of surface hardness of the two denture base RDS is shown in Table 3. The hardness measurement before fixing RDS to dentures demonstrated the statistically significant difference (*P* < 0.05); with heat cure AR recorded higher hardness mean value than TR (18.46 and 13.65 respectively). With denture insertion and utilising cleansing tablets for three months a slight decrease in mean values were recorded (14.90 & 11.81) for AR and TR respectively, however, it was not statistically significant (*p* > 0.05).

## Discussion

Denture base surface properties are of peculiar importance as they affect the denture longevity during the function. Surface roughness and hardness have been investigated utilising different in-vitro methods. All these techniques provide valuable information regarding the mechanical properties of the materials tested, however, none of in vitro techniques can expose the tested materials to conditions similar to that of the oral environment (*in vivo*) such as pH and temperature variations [20].

Hence, the association between the results of in vitro methods and clinical studies are expected to show some discrepancies [21]. In the current study, therefore, a sampling technique developed by Avon et al, [22] was modified and utilised to provide reliable information about the influence of denture cleanser coupled with that of oral cavity on both surface roughness and hardness of two denture base materials [23].

Denture hygiene and disinfection have been recommended as an essential practice for preventing cross-contamination and the maintenance of a healthy oral mucosa. It has been pointed out that some disinfection methods may have unfavourable effects on denture base resins [24, 25]. The surface roughness of any denture base material influences microbial colonisation and biofilm formation [26, 27]. Furthermore, roughness causes denture discoloration and it may predispose patient discomfort. The surface roughness (Ra) of 0.2 μm was reported to be a clinically acceptable value, where no further decrease in plaque accumulation is anticipated indenture prostheses as reported in the literature [28-31]. In this study, maxillary dentures were designated with retrievable removable discs (RDS) from two materials: conventional acrylic resin and thermoplastic one. Both RDS showed roughness within the acceptable value (Ra 0.2 μm) prior to fixing RDS to dentures. Despite the difference in the chemical composition and curing method, the accurate laboratory procedures and following manufacturers’ instructions aiming to achieve the smooth surface quality may account for this nearly similar roughness values.

Conversely, after three months of using cleansing tablets, retrieval of RDS revealed an apparent matching increase in roughness (Ra) above the acceptable value. This increase in roughness might be attributed to possible changes occurring in RDS polymer materials as a consequence of the coupled effects of oral environment and the use of denture cleanser. These findings are in agreement with a previous study which reported that effervescent cleansing tablets increase the surface roughness [32].

Furthermore, the results of this study demonstrated that the conventional acrylic resin RDS presented a higher hardness than thermoplastic resin before fixing RDS to dentures. This probably due to the difference in chemical composition were; high monomer-polymer content, presence of methyl-methacrylate monomer and cross-linking agents are influencing factors for the better surface hardness of the conventional acrylic resin [33].

Interestingly, a comparable decrease in the hardness for both RDS materials after retrieval of specimens was evident. This result is in accordance with previous studies demonstrated reduction of hardness after using different disinfection methods on different denture base materials [32, 34, 35]. Several reasons may explain the previous results as water absorption during disinfection may act as a plasticizing agent, which permits relaxation of stresses occurred during processing and consequently, hardness is lowered [36, 37]. It was reported that repeated exposure of the dentures to disinfectant solutions may alter their physical properties. Moreover, some chemical constituents of the disinfectants may result in softening and degradation of the surface layer of denture resin. Another explanation is that denture disinfectant liberates oxygen resulting in hydrolysis and disintegration of the polymerised resin [24, 38, 39].

In conclusion, within the limitations of this in vivo study, it could be concluded that denture cleansers affect the surface roughness and hardness concurrently with oral condition variations, which will consequently influence the durability and satisfaction of complete denture wearers.
